# Minimally invasive percutaneous screw internal fixation under robot navigation for the treatment of a hamate bone fracture

**DOI:** 10.1186/s12891-023-06917-6

**Published:** 2023-12-01

**Authors:** Fang Jie, Zhu Hui, Zheng Dawei, Liu Guiqian, Shi Rongjian, Qi Weiya

**Affiliations:** Department of Hand Surgery, Clinical Anatomy Laboratory, Xuzhou Renci Hospital, No. 11 Yangshan Road, Jinshanqiao Street, Xuzhou City, Jiangsu, 221004 Jiangsu People’s Republic of China

**Keywords:** Hamate fracture, Robot navigation, Minimally invasive

## Abstract

**Purpose:**

Hamate fractures are rare fractures of the wrist and there is still no consensus on the optimal treatment for these fractures, especially hook of hamate fractures. Herein, the authors present a case study of a series of patients who were treated with closed reduction and minimally invasive percutaneous fixation under robot navigation.

**Methods:**

This retrospective study reviewed 14 patients who had nondisplaced or minimally displaced hamate fractures on computerized tomography images and were treated using the treatment in our centre from November 1, 2019, to October 31, 2022. At the final follow-up, the flexion-extension and radial-ulnar range of motion of the wrist were measured, and the grip strength and pinch strength were measured. The pain of the wrist was assessed using the visual analogue scale (VAS). The Mayo wrist score reflected the recovery of the wrist.

**Results:**

The mean total operative duration was 40.1 min. All the fractures showed union at a mean of 3.0 months. At a mean follow-up of 23.3 months (range 6–36 months), the mean VAS score was 0.7, the average Mayo wrist score was 95, and the mean pinch strength and grip strength were 11.3 and 38.7 kg, respectively. The flexion-extension arc was 138.3°, the mean radial and ulnar deviation arc was 63.8°, and the mean pronation-supination arc was 172.3°. And the time of return to the original occupation was mean 4 months (3~6 months). There were no complications, such as infection or nerve paralysis.

**Conclusions:**

This study suggests that nondisplaced or minimally displaced hamate hook fractures can be successfully treated by closed reduction and internal fixation with a headless compression screw with the assistance of robot navigation, and the small fragment of fracture can be accurately fixed with minimal iatrogenic injury.

**Supplementary Information:**

The online version contains supplementary material available at 10.1186/s12891-023-06917-6.

## Introduction

Hamate fractures are rare, account for approximately 2%~4% of all carpal fractures, and are common in young adults or athletes because such fractures are caused by direct trauma to the hand, resulting in direct transfer of force onto the bone, especially during sports in which the position of the ulna is deviated at the wrist during the establishment of a power grip as in golf, baseball and racquet sports [1–3]. It may also be associated with a stress fracture caused by repetitive overload [4]. According to Milch, hamate fractures can occur in the hook or body of the bone, and hamate hook fractures are more common than hamate body fractures [5, 6].

Such fractures are difficult to diagnose using standard anteroposterior and lateral radiographs. The diagnosis can be made early with a carpal tunnel view and computed tomography (CT) [2, 7]. Delayed diagnosis and treatment of a hamate hook fracture can result in nonunion, which may be associated with chronic pain on the ulnar side of the palm, which can be aggravated by grasping and may lead to other complications, such as rupture of the flexor tendon of the ring or little finger and ulnar nerve dysfunction [7, 8]. Early diagnosis and early excision are recommended for athletes to minimize morbidity and promote an early return to physical activity [2]. The hook of the hamate bone articulates the radial border of the Guyon canal and the ulnar border of the carpal tunnel. It also serves as an attachment site for the short flexor and opponens muscles of the little finger, transverse carpal ligament, and perihamate ligament, which provides stability. The base of the hook works as a pulley for the flexor tendons of the fourth and fifth digits [1, 2, 9]. A biomechanical cadaveric study indicated that the induction of grip strength is positively correlated with the level of resection of the hook [10]. However, the optimal treatment for an acute hamate fracture has not been identified.

The purposes of this study were to present our surgical procedure and to assess the clinical and radiological outcomes of minimally invasive percutaneous headless compression screw internal fixation with TiRobot navigation for an acute hamate fracture in a nonathletic population in a larger patient on the basis of the previous study [6].

## Materials and methods

### Ethical approval

The study was conducted in accordance with the Declaration of Helsinki and was approved by the ethics committee of authors’ Hospital (RC201911001). Informed consent was obtained from all the patients preoperatively.

### Patient information

The medical records of fourteen consecutive patients with an acute hook of hamate fracture who were treated via minimally invasive percutaneous headless compression screw internal fixation under TiRobot navigation in our centre were retrospectively reviewed between November 2019 and October 2022. Eleven men and three women, with a mean age of 39.1 years (23–66 years). The patients arrived at our centre 2.8 days after the injury (range, 0.06 to 12 days). The mechanisms of injury included falls in ten patients, accidental injuries in two patients and bruising injuries in two patients; ten of them were in the right hand, and four were in the left hand [Table 1].

**Table 1:** Preoperative Characteristics

Case	Sex	Age *(yr)*	Time to Surgery (days)	Cause of injury	Involved hand	Combined injury	Time of the operation (minute)	Bone union time (month)
1	M	26	0.3	Fall	Right	Triquetrum fractured	45	3
2	M	50	3	Fall	Right		40	4
3	M	32	4	Fall	Right	Capitate bone	32	3
4	M	23	7	Fall	Right	Radial fracture, third and fourth metacarpophalangeal fracture	45	3
5	M	37	3	The bruise injury caused by heavy objec	Left	Radial and ulnar fracture, the first metacarpophalangeal fracture	40	2.5
6	M	34	1	The bruise injury caused by heavy objec	Left	Radial and ulnar fracture	55	2.5
7	F	66	0.08	Fall	Left	Fourth metacarpophalangeal fracture and dislocation	45	3.5
8	M	31	6	Fall	Right		30	3
9	M	33	0.08	Fall	Right	Fourth and fifth metacarpophalangeal dislocation	40	3
10	M	26	0.79	Fall	Left	Scaphoid, capitate, lunate, trapezium fracture, Fourth and fifth metacarpophalangeal dislocation	35	3
11	M	29	12	Fall	Right	Fourth metacarpophalangeal fracture and dislocation	35	2.5
12	M	47	1	Fall	Right	Fourth metacarpophalangeal fracture and dislocation	40	3
13	F	57	0.06	Traffic accident injuries	Left	Fourth metacarpophalangeal fracture, distal radio and ulnar fracture	45	3
14	F	56	1	Traffic accident injuries	Right	Scaphoid and pisiform fracture	35	3.5
Mean		39.1	2.8				40.1	3.0

### Surgical indications

Minimally invasive percutaneous screw internal fixation with robot navigation is indicated for a fresh, nondisplaced or minimally displaced hook of hamate fracture. This method is contraindicated for hook of hamate fracture nonunion, fractures with a delayed presentation, fibrous nonunions and displaced fractures.

### Facility information

TiRobot is a robotic navigation system (TINAVI Medical Technologies, Beijing, China) with an optical tracking system, a stereotactic robotic arm with six degrees of freedom, and a surgical planning workstation. It has been used with a three-dimensional (3D) fluoroscopy unit (ISO-C3D, Siemens, Erlangen, Germany) for 3D image capture and K-wire guidance positioning [11]. The optical tracking device is the eyes of the TiRobot, which consists of an infrared stereo camera and two reference frames. One reference frame is a patient reference, which is stably and securely fixed onto a customized and radiolucent wrist positioning jig, and the other is the robotic reference frame, which is attached to the robotic arm to identify and collect the position information of the wrist relative to the robotic arm. They are the key components of robot navigation in that they are used to guide the robot to the planned position.

**Fig. 1:**
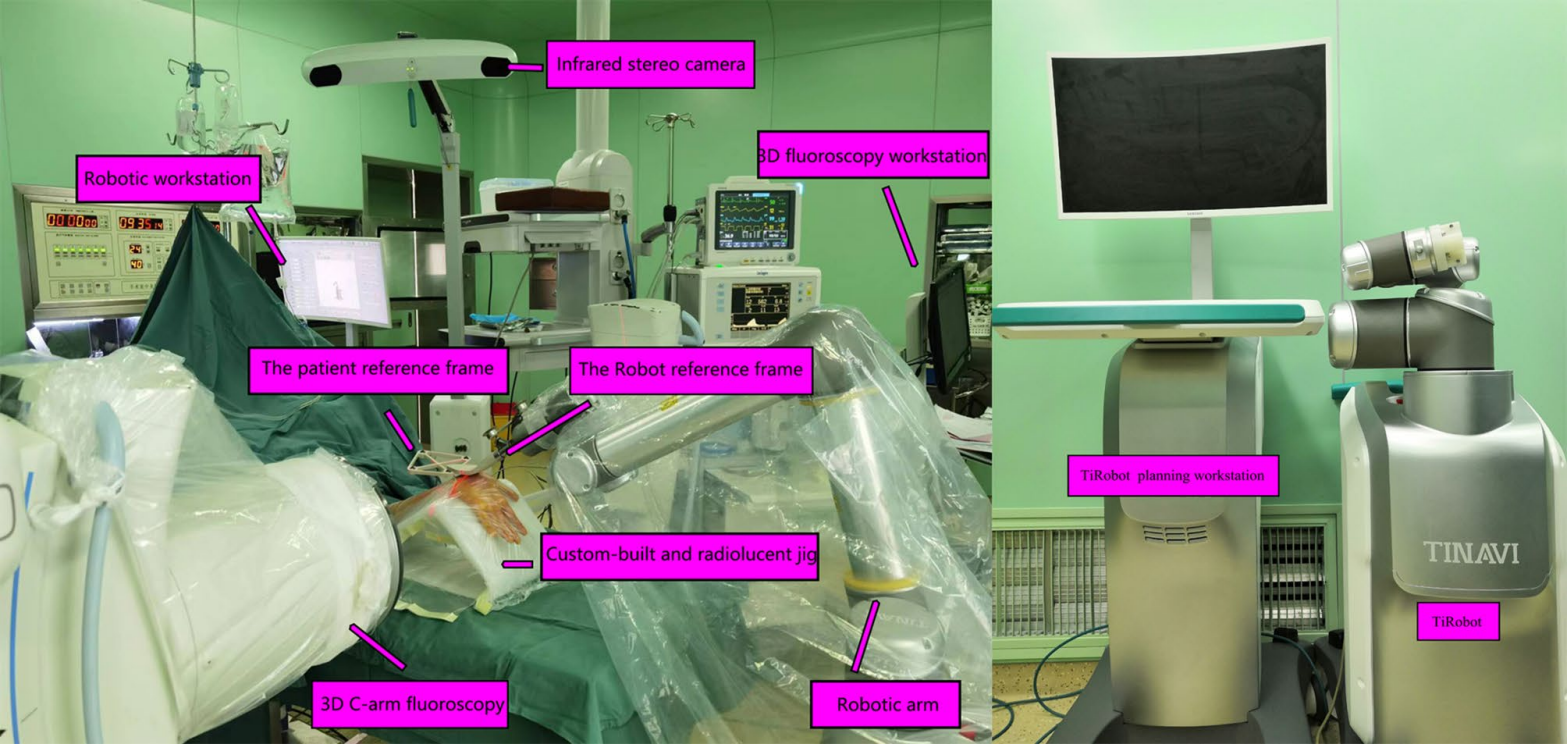
TiRobot navigation system

The 3D fluoroscopy unit is the image acquisition system that is used to capture and process the axial, coronal, and sagittal planes of the fractured bone. Then, the images are delivered to the TiRobot workstation to construct a 3D volumetric image for intraoperative planning, and its components include a 3D C-arm and a C-arm reference tracker, which can circumferentially scan images around the target. The position was confirmed by fluoroscopy, while the C-arm and the reference frames were appropriately positioned, after which an automated registration scan was performed, and the images will be circumferentially captured around the patient’s wrist in fixed angular steps [Fig. 1].

### Surgical procedure

**Fig. 2:**
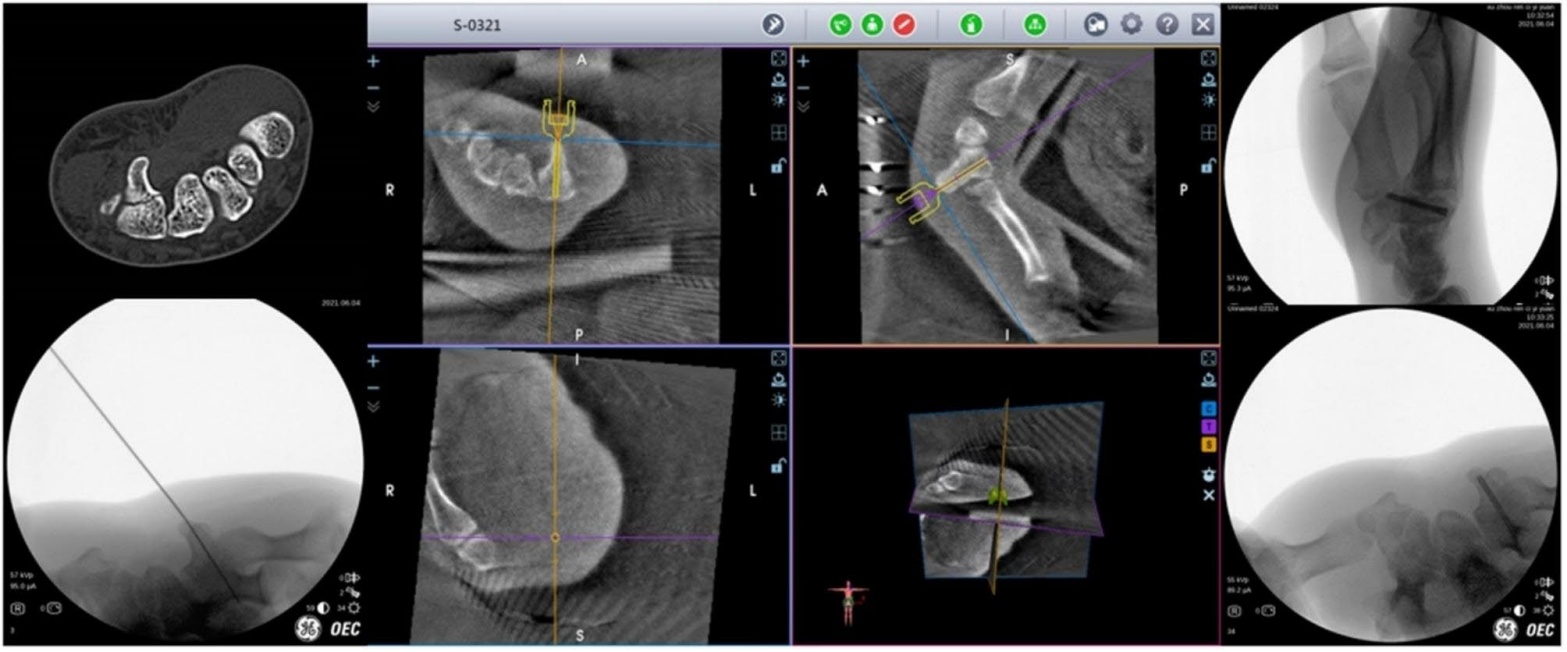
The preoperative CT images of the patient showed a hook of hamate fracture. Guidewire placement is achieved by using TiRobot navigation, and fracture reduction is achieved by using fluoroscopy. A headless cannulated compression screw is then used to rigidly secure the hook of the hamate fracture through a dorsal approach

Closed reduction was performed under fluoroscopy before the wrist was extended and securely affixed to a custom-built, radiolucent jig and the table in necessity. While the fragment was optimally reduced, a K-wire guidance was temporarily inserted into the hook of the hamate fracture by the assistant in necessary. The wrist and table must not move during the operation. The details of the surgical procedure are described in the manuscript [6]. The key to the treatment is the percutaneous placement of a Kirschner guidewire along the central axis of the reduced hook of the hamate bone fracture. Screws placed along the central axis have been reported to increase the rate of healing, increase the stiffness of fixation and reduce the risks of thread penetration and adjacent tissue injury [11]. Guidewire placement is achieved by using TiRobot navigation, while the tip of the guidewire was penetrated the dorsal soft tissue, and the hand is removed from the operating rig to proceed with setting the screw. A headless cannulated compression screw (Hopromed, Jiangsu Hopromed Medical Technology Co., LTD, China) is then used to rigidly secure the hook of the hamate fracture through a dorsal approach to reduce the risk of injuring the adjacent vessels and nerves. [Fig. 2].

### Hamate imaging

**Fig. 3:**
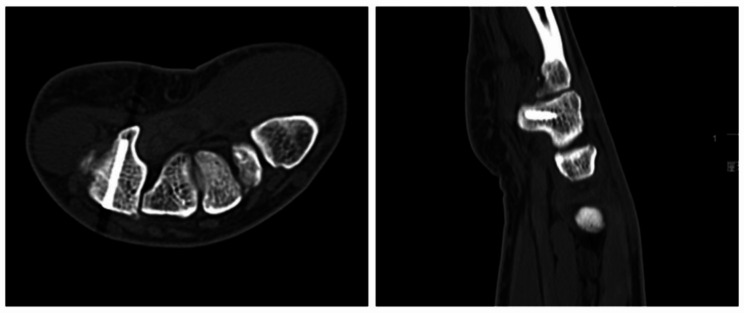
The CT images showed that the hamate hook fracture had healed at three months

The carpal bone position was captured with a three-dimensional (3D) fluoroscopy unit (ISO-C3D, Siemens, Erlangen, Germany) through posterior-anterior and lateral radiographs. Then, a 3D image was captured through ring scanning and transmitted to the TiRobot workstation. The entry point, trajectory, and length of the headless compression screw were simulated and planned in a visualized TiRobot workstation image screen using 3D volumetric image data. Then, a 0.8 mm guide pin was drilled into the hook from palmar to dorsal, and a less than 0.5 cm incision was made in the opisthenar to allow placement of the headless compression screw. Subsequently, the screw was inserted into the hook through the trajectory. The position and length of the screw were assessed under fluoroscopy. [Fig. 3]

### Postoperative management and follow-up

The sutures and self-adhesive bandage were removed 2 weeks postoperatively. Physical rehabilitation of the fingers of the hand, including active and passive rehabilitation, was started when the wound pain was relieved. Then, physical rehabilitation, including active and passive rehabilitation, of the wrist was started.

A senior hand surgeon who was not involved in the surgery evaluated the postoperative position, length and trajectory of the headless compression screw by comparing the intraoperative 3D C-arm image and the postoperative CT image.

The patient’s condition was reviewed in our department at 1 month, 2 months, 3 months, 6 months and the final follow-up, and additional follow-up is arranged if indicated. Union of the hook of the hamate was confirmed by CT scan, while the fracture line of the fragment completely disappeared postoperatively. At the final follow-up, the flexion-extension and radial-ulnar range of motion of the wrist was measured by a goniometer. The grip strength and pinch strength were measured and compared with the contralateral wrist at the final follow-up. Visual analogue scale (VAS) scores range from 0 (no pain) to 10 (severe pain), and the results are divided into three classes (good < 5, fair 7 − 5, poor 10 − 8) used to assess the pain of the wrist. The Mayo wrist score reflected the recovery of the wrist.

## Results

### Operating time

The average total operative duration was 40.1 min (range 32~55 minutes), which included the time for patient and equipment positioning and the time for registration scanning; the surgical time was 21.5 min (range 15~28 minutes), which included the time from simulating and planning the screw trajectory on the TiRobot workstation to skin closure. Only a single guide wire was inserted into the hook of the hamate bone of all the patients. According to the TiRobot workstation parameter, the length of the screw was chosen ranged from 1.4 to 2.2 cm which is 2–4 mm shorter than the measurement provided by the TiRobot workstation. The main point is the screw can’t protrude out of the bone dorsal or palmar cortex, and the diameter all are 2.5 cm.

### Accuracy of screw placement during surgery

Intraoperative X-ray and 3D volumetric image data and postoperative X-ray and CT scanning showed that the actual position of the implanted screw was the same as the planned position in all the patients. The length of the headless compression screw was exactly the same as that on intraoperative planning from the workstation length. Accurate positioning can be determined, and the dimensions and trajectories of the screws can be successfully acquired. There was no cortical violation following screw placement via the dorsal or palm plane in any of the patients. There were no intraoperative or postoperative complications.

### Fracture healing time and functional recovery

**Fig. 4:**
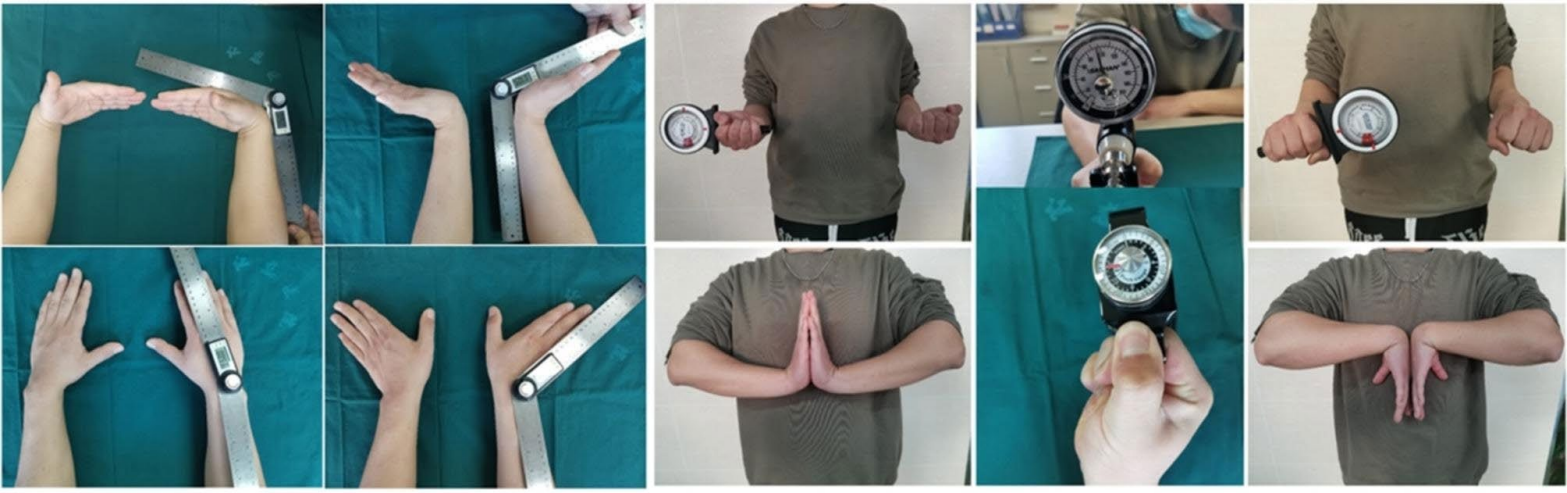
Patient, male, 24 years old, the right hook of the hamate in nondisplaced fracture, treated with closed reduction and minimally invasive percutaneous headless compression screw internal fixation with TiRobot navigation. After 24 months of follow-up, the function and appearance had recovered satisfactorily

In all the patients, the hook of the hamate fracture was primarily healed within a mean of 3.0 months postoperatively. Fracture union and whether the position of the screw was in its original implanted position was confirmed by CT in all the patients. The mean follow-up time was 23.3 months (range 6–36 months). At the final follow-up, the mean VAS score was 0.7 (0~3), the average Mayo wrist score was 95 (80~100), twelve patients had excellent recovery and two had good recovery, and the mean grip strength and pinch strength were 11.3 kg (5.5~15 kg) and 38.7 kg (25.2~48 kg), respectively. The flexion-extension arc was 138.2°, the mean radial and ulnar deviation arc was 63.8°, and the mean pronation-supination arc was 172.3°. [Fig. 4] And the time of return to the original occupation was mean 4 months (3~6 months). There were no complications, such as infection or nerve paralysis, [Table 2].

**Table 2:** Data of the final follow-up

case	Follow-up time (months) 1	VAS score	The radial-ulnar deviation (°)	The palmar flexion dorsal extension (°)	The forearm pronation- supination (°)	The pinch strength (Kg)	The grip strength (Kg)	The modified Mayo wrist score	Time of return to the original occupation (months)
1	17	0	65.7	156.9	180	12	38	100	3
2	22	1	49.5	125.2	180	14	42.6	100	4
3	34	0	72.7	153.8	170	9.5	38.4	95	3
4	28	0	69.4	132.9	170	12	44	100	3.5
5	30	1	75.6	149.2	168	9	36.4	95	4
6	20	0	66.3	143.2	180	15	46.8	100	3.5
7	17	2	53.3	120.2	175	7.5	35.2	85	5
8	13	0	65.2	157.3	160	8	34	95	4
9	33	0	59.1	161	175	15	45.6	100	5
10	36	0	64.8	124.4	180	13.5	42	100	3
11	28	0	58.6	166.6	170	15	48	95	3
12	2	1	75	129.7	180	12	39.4	95	5
13	2	1	54	118	180	5.5	28	80	4
14	35	3	90.3	122.6	165	6	29.6	90	6
Mean	23.3	0.7	63.8	138.2	172.3	11.3	38.7	95	4

## Discussion

In our case series study, we found that TiRobot navigation can accurately position and determine the dimensions and trajectory of the screws intraoperatively, which has been confirmed by postoperative radiography and CT scans. All the hamate hook fractures were completely healed in a mean of 3 months following closed reduction and percutaneous internal fixation in our centre without any accidental complications. The mean operative time was 40.1 min (range, 32~55 minutes). The Mayo wrist score of the injured wrist was 95, twelve patients had excellent recovery and two had good recovery, and the outcomes were satisfactory. The wrist outcomes were superior to those associated with radio- and ulnar fractures. The patient returned to his normal life and original occupation within a mean of four months postoperatively.

Although it has been reported in the literature that the incidence of a hamate hook fracture is very low in nonathletes who present to the emergency department annually, the incidence may be higher than previously thought, [12] and the treatment is a challenge [13]. However, to date, there is still no consensus on the optimal treatment. Numerous methods have been reported in the literature, such as conservative treatment, ORIF, and excision [14, 15]. However, Scheufler reported that the nonunion rate is more than 83.3%; [15] furthermore, the rate of painful nonunion is 90 to 100% following treatment failure and other complications have been described [16, 17]

Surgical excision was considered a standard treatment for patients suffering from symptoms of chronic and established nonunion of a hamate hook fracture [18]. A retrospective study showed that surgical excision of a hamate hook fracture could relieve pain and restore normal function, leading to high satisfaction rates in high-level amateur athletes [19]. Nevertheless, the hamate hook acts as a pulley, which plays an important role in the function of the flexor tendon of the fourth and fifth fingers and has some biomechanical advantages, and the power grip may be decreased after hamate hook excision because of changes in tendon force [20–22]. Additionally, there is a risk factor for damage to adjacent vital structures, including the motor branch of the ulnar nerve, the ulnar digital nerve of the litter finger, and the flexor tendons of the ring and little fingers [23].

Hook plates have been successfully used to treat nonunited hamate hook fractures. However, open fixation increases the risks of complications and injury to the ulnar artery trifurcation, ulnar nerve, and flexor tendon [24] and has a longer recovery time and higher risk of nonunion [25]. There are also risks associated with the treatment of dorsal percutaneous cannulated screw fixation [26, 27] in that it is difficult to percutaneously insert a k-wire or fix a screw in the optimal central axis of the hook of hamate bone, which has an average dimension of 1.3 by 1.0 by 0.5 cm [28]. Hook fractures are at high-risk of avascular osteonecrosis and nonunion [29]. Methods to reduce the risks of iatrogenic trauma and injury to the adjacent vital tissue are needed. The goal of treatment is the recovery of the normal anatomical structure. The initial idea of effective and accurate location of surgical instruments combined with anatomical structures in the human body interoperative can be traced back to the late nineteenth century [30].

In our series, the average time from injury to surgery was 2.8 days; most of the patients were not immediately diagnosed and treated upon presentation to the emergency department, however only three of the patients came to our consulting room and consented to undergo emergency surgery for their injury. Computed tomography has been proven to have 100% sensitivity, 98.4% specificity, and 97.2% accuracy in diagnosing a hamate hook fracture [31]. All of our patients were diagnosed by CT scan. A new commercially available robot associated with the 3D fluoroscopy unit for surgical navigation called TiRobot (TINAVI Medical Technologies Co., Ltd., Beijing, China) was produced for accurate and safe navigation in planning the screw trajectories and the optimal position of the implants [6, 11, 32]. 3D reconstructed CT images have been proven to provide a more accurate and insightful illustration for surgical planning [6]. TiRobot navigation and the advent of 3-D fluoroscopy offered further improvements in accuracy [31]. The guidewire was successfully inserted at the first attempt in all of the patients, without causing any iatrogenic injury to the adjacent tissues. Because the diameter of the headless screw is larger than the guidewire, and the rate of the risk of injuring the adjacent vessels and nerves is increased. Therefore, we inserted the guidewire from the volar side and place the headless screw from the dorsal side. The active wrist motion of twelve patients was completely restored, and that of the other two patients with distal radial and ulnar fractures was impaired.

The treatment provided depends on the facility, and the cost of the TiRobot system is high. The widespread application of the technique is limited by the popularity of the facility. Moreover, the learning curve of designing and stimulating the entry point and path is easy in a visualized TiRobot workstation image screen. The small sample size is a limitation of our study. A large, randomized, prospective study or a comparison study of different treatments should be performed to assess the superiority and benefits of robot navigation in the future.

Because the TiRobot has the potential to solve the core issue of pedicle screw instrumentation, we treated the small fragment of a hamate hook fracture under robot navigation. The results show that the technique is promising, and the patient’s functional recovery indicate that the technique provides effective fracture management, especially for nondisplaced or minimally displaced hook fractures.

## Conclusions

In conclusion, the treatment for nondisplaced or minimally displaced hamate hook fractures by closed reduction and internal fixation with a headless compression screw with the assistance of robot navigation, and the small fragment of fracture can be accurately fixed with minimal iatrogenic injury, and the outcomes exhibit that bone healing time and the bone union rate is satisfactory.

### Electronic supplementary material

Below is the link to the electronic supplementary material.

Supplementary Material 1

## Data Availability

The datasets supporting the conclusions of this article are included within the article. Raw data can be requested from the corresponding author.

## Abbreviations

VAS: visual analogue scale
CT: computed tomography
3D: three-dimensional
ORIF: open reduction and internal fixation
