# Supplementary material for: Minimally invasive percutaneous screw internal fixation under robot navigation for the treatment of a hamate bone fracture
Source: BMC Musculoskelet Disord. 2023 Dec 1;24:929. doi: 10.1186/s12891-023-06917-6 (PMC10691140; doi:10.1186/s12891-023-06917-6)
Supplement: Supplementary file 1 — Supplementary Material 1 [file 12891_2023_6917_MOESM1_ESM.docx]

Supplementary Material

*Case presentation*

A 23-year-old man who fell and injured his right wrist sustained a hook of the hamate fracture. He came to our centre 8 hours after sustaining the injury and underwent closed reduction and internal fixation with a headless compression screw with the assistance of robot navigation. At three months postoperatively, CT of his wrist showed that the fracture was healing [Fig. 3]. At the final follow-up, his VAS score was 0, his Mayo wrist score was 100, he showed excellent recovery, and his grip strength and pinch strength were 12 kg and 44 kg, respectively. The flexion-extension arc was 132.9°, the radial and ulnar deviation arc was 69.4°, and the pronation-supination arc was 170°. There was no infection or nerve paralysis [Fig. 4].
